# Quantification of 3‑chloro-7‑hydroxy-4-methylcoumarin (CHMC) in urine as a biomarker of coumaphos exposure by high-performance liquid chromatography-fluorescence detection (HPLC-FLD)

**DOI:** 10.1016/j.mex.2025.103171

**Published:** 2025-01-13

**Authors:** Zachary G. Robbins, Cynthia A. Striley, Lee Wugofski

**Affiliations:** a Chemical and Biological Monitoring Branch, Health Effects Laboratory Division, National Institute for Occupational Safety and Health, 1090 Tusculum Ave, Cincinnati, OH 45226, USA; b Federal Occupational Health, Program Support Center, Assistant Secretary for Administration, Office of the Secretary, U.S. Department of Health and Human Services

**Keywords:** Coumaphos, 3‑chloro-7‑hydroxy-4-methylcoumarin, Chlorferon, HPLC-FLD, Organophosphates, Pesticides, Urine analysis, Biomonitoring, Quantification of 3‑chloro-7‑hydroxy-4-methylcoumarin (CHMC) in urine as a biomarker of coumaphos exposure by high-performance liquid chromatography-fluorescence detection (HPLC-FLD)

## Abstract

The organophosphate pesticide coumaphos is used to control Cattle Tick Fever carried by multiple species of ticks and is a known hazard for workers treating livestock. The USDA Cattle Fever Tick Eradication Program requires regular blood draws to measure depressed cholinesterase levels as biomarkers of effect of long-term coumaphos exposure, however, the gap between blood draws may miss intermittent high exposures. Urine biomonitoring can supplement blood draws, offering personnel a sensitive and cost-effective method to monitor short-term exposures. Our objective was to improve and validate a previously published method to analyze the coumaphos metabolite 3‑chloro-7‑hydroxy-4-methylcoumarin (CHMC). Urine samples were hydrolyzed with glucuronidase and then extracted prior to analysis with high-performance liquid chromatography-fluorescence detection. Calibration curves were linear over a wide CHMC range (0.49 – 250.07 ng/mL) with a method detection limit of 0.06 ng/mL. This research will help establish an accessible urine biomonitoring method for assessing coumaphos exposures.

• The modified bioanalytical method maintained high sensitivity and specificity while reducing duration of the sample treatment steps and the chromatographic program.
• Method validation tests followed the acceptance criteria guidelines in the NIOSH Manual of Analytical Methods.
• CHMC levels were measured in workers exposed to coumaphos during livestock treatment.

## Graphical abstract

**Figure:**
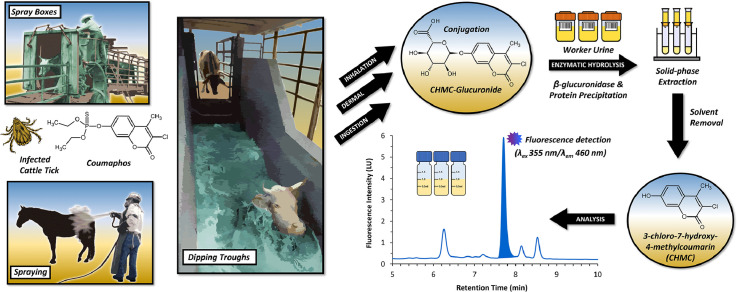

Specifications table

**Table:** 

Subject area:	Chemistry
More specific subject area:	Pesticide biomonitoring
Name of your method:	Quantification of 3‑chloro-7‑hydroxy-4-methylcoumarin (CHMC) in urine as a biomarker of coumaphos exposure by high-performance liquid chromatography-fluorescence detection (HPLC-FLD)
Name and reference of original method:	Evaluation of coumaphos exposure among tick eradication workers, **published in:** Thomas, G. A., Delaney, L. J., Mueller, C., & Page, E. (2010). Evaluation of coumaphos exposure among tick eradication workers. *Journal of occupational and environmental medicine, 52*(2), 131–136. https://doi.org/10.1097/JOM.0b013e3181cd7e80.
Resource availability:	NA.

## Background

The US Department of Agriculture (USDA) Animal and Plant Health Inspection Service (APHIS) and the Texas Animal Health Commission (TAHC) lead the Cattle Fever Tick Eradication Program (CFTEP) in a permanent quarantine zone near the Texas-Mexico border, overseeing more than 400,000 cattle operations to control the North American cattle tick (*R. annulatus*) and the Asian Blue tick (*R. microplus*) [[1], [2], [3], [4], [5], [6]]. These ticks transmit Cattle Tick Fever (bovine babesiosis) to cattle and other ungulates (i.e., horses), which has a 70–90 % mortality rate and does not have a universally effective vaccine [1-4]. The organophosphate pesticide coumaphos (CAS No 56–72–4) was first registered in 1958 to control tick infestations and it has been heavily restricted since 1996 [2,[5], [6]]. An ongoing exception has been for livestock treatment in the Texas quarantine zone due to the estimated economic impact of greater than $3 billion in losses [4,5]. Coumaphos is classified as a highly hazardous pesticide (Class Ib) by the World Health Organization (WHO) and the US Environmental Protection Agency (EPA) [[7], [8], [9]].

CFTEP animal health technicians may potentially be exposed to coumaphos during varied work tasks and often do not wear adequate personal protective equipment due to ambient weather conditions and risk of heat stress [[2], [6]]. Technicians mix coumaphos solution, apply coumaphos to livestock with dipping vats, spray-booths, and hand-sprayers, conduct physical examinations of livestock, and clean equipment [[2], [6]]. Organophosphate pesticide exposures are associated with cholinergic health effects emanating from the inactivation/inhibition of cholinesterase in the nervous system [[3], [10]]. Acute cholinergic effects from organophosphate pesticide poisoning may include nausea, headaches, drowsiness, confusion, muscle spasms, circulatory or respiratory depression, and in rare cases respiratory failure [7]. Repeated exposures may lead to exacerbated acute effects and are also linked to chronic effects such as memory loss, speech impairment, neuropsychiatric disorders, and allergic asthma [[7], [11]].

**Fig. 1:**
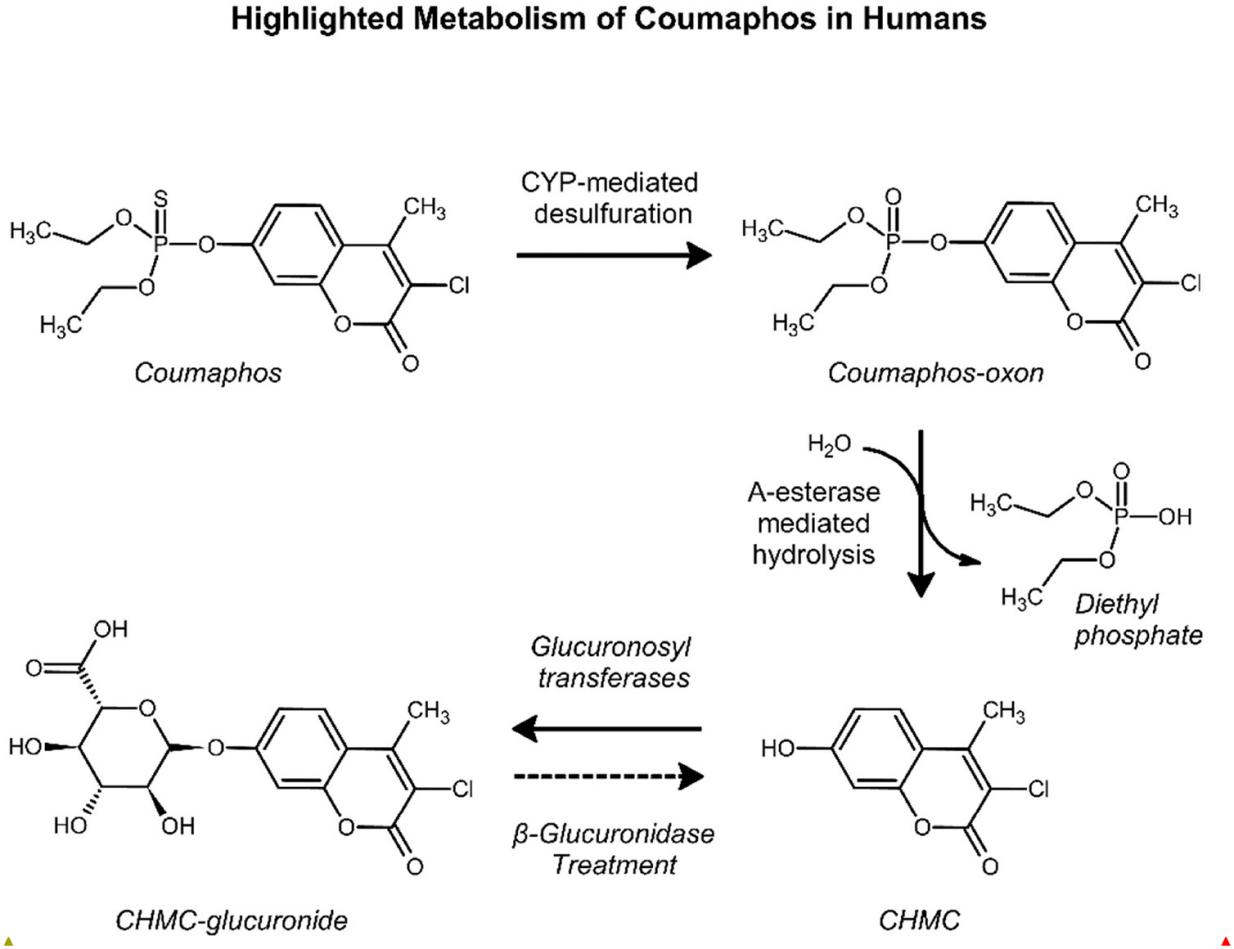
Coumaphos is metabolized by cytochrome P450 through oxidative desulfuration to form the coumaphos-oxon, which is then catalytically hydrolyzed by A-esterase (phosphotriesterase) enzymes to form CHMC and diethyl phosphate [25]. Glucuronosyltransferases facilitate the conjugation of CHMC into its corresponding glucuronide which is excreted in urine. Prior to sample extraction and analysis, urinary CHMC-glucuronide is deconjugated via β-glucuronidase.

USDA APHIS requires CFTEP workers to undergo regular venous blood draws to monitor red blood cell acetylcholinesterase (RBC AChE) and plasma pseudocholinesterase (PChE) activity as biomarkers of effect of coumaphos exposure [[2], [5], [6]]. If an individual's RBC AChE or PChE falls below 75 % of baseline, the individual must be withdrawn from coumaphos related tasks until levels return to an acceptable range [2]. Most CFTEP workers treat livestock in rural and rugged locations, complicating the logistics of performing blood draws to monitor cholinesterase. Urine biomonitoring supplements cholinesterase monitoring because it can be collected on-site and is an effective tool for observing short-term exposures between blood draws [[2], [3], [12]]. Coumaphos is metabolized to 3‑chloro-7‑hydroxy-4-methylcoumarin (CHMC; also known as chlorferon) and excreted in urine as a glucuronide conjugate (see Fig. 1) [[2], [13], [14], [15], [16]]. Thomas et al. [2] previously developed a high-performance liquid chromatography-fluorescence detection (HPLC-FLD) method to analyze CHMC in urine collected from CFTEP workers. While liquid chromatography-mass spectrometry (LC-MS) is often used to analyze biomarkers of organophosphate pesticide exposures [[14], [17], [18], [19], [20], [21], [22]], HPLC-FLD is a low-cost and sensitive alternative for naturally fluorescent organophosphate metabolites such as CHMC. Our goal was to improve and validate the previously published method following the National Institute of Occupational Safety and Health (NIOSH) guidance [[2], [23]] and quantify CHMC levels in urine collected from CFTEP workers in a collaborative exposure assessment study with USDA APHIS and Federal Occupational Health (FOH).

## Method details

### Chemicals and reagents

Ammonium acetate (ACS grade, ≥97 %), sodium acetate (anhydrous), acetonitrile (Optima™ grade, ≥99.9 %), methanol (Optima™ grade, ≥99.9 %), and formic acid (Optima™ grade, ≥99.0 %) were obtained from Fisher Scientific (Pittsburgh, PA, USA). 3-Chloro-7‑hydroxy-4-methylcoumarin (CHMC; 97 %) was obtained from Sigma-Aldrich, Inc. (St. Louis, MO, USA). IMCSzyme® beta-glucuronidase reagent and IMCSzyme® rapid hydrolysis buffer were obtained from IMCS (Irmo, SC, USA). Deionized water (Type I) was produced by a Milli-Q® Integral 15 water purification system (MilliporeSigma, Burlington, MA, USA). Pooled urine from unidentified males (not filtered; not viral tested) was obtained from BioIVT (Westbury, NY, USA).

### CFTEP study population

Three spot urine samples (pre-shift, post-shift, and next-morning) were collected in the summer of 2019 from 19 study participants for a total of 56 urine samples. One worker did not collect a next-morning sample. Participants were provided oral and written instruction for proper collection of urine samples. Spot urine samples were collected in sterile 250 mL plastic cups as directed by USDA APHIS and FOH personnel. Cups were marked with pre-printed labels indicating worker ID, unique ID, time, date, and location. After collection, urine samples were immediately refrigerated on-site and frozen before shipment. Frozen urine samples were shipped on dry ice and stored in a freezer (−60 °C) until needed. Prior to sample treatment and analysis, urine was allowed to thaw to room temperature and specific gravity was measured with a Fisherbrand handheld digital clinical refractometer (Fisher Scientific, Pittsburgh, PA). Specific gravity levels were provided to USDA APHIS and FOH but are not reported in the manuscript.

### CHMC standard preparation

The bioanalytical method was developed using the method evaluation criteria recommended by the NIOSH Manual of Analytical Methods, 5th Edition [23]. CHMC standards were prepared as follows. A stock solution of CHMC (approximately 500.0 µg/mL) was prepared in a 25 mL volumetric flask with 10 mmol sodium acetate in deionized water (pH 5.3). An intermediate calibration standard (approximately 25.0 µg/mL) was prepared by adding approximately 500 µL of the stock solution into a 10 mL volumetric flask and bringing the flask to volume with 10 mmol sodium acetate in deionized water. Spiking calibration standards (N = 10) were prepared by serial dilution in 4 mL borosilicate glass vials. The highest spiking calibration standard was prepared by adding 1 mL of the intermediate calibration standard into 1 mL of 10 mmol sodium acetate in deionized water. The highest spiking calibration standard was then serially diluted (1:1 v/v) with 1 mL of 10 mmol sodium acetate in deionized water to obtain the following concentrations: 0.02, 0.05, 0.10, 0.20, 0.39, 0.78, 1.56, 3.13, 6.25, and 12.50 µg/mL. Urine standards for sample treatment and analysis were prepared by adding 20 µL of each spiking calibration standard into 980 µL pooled control urine. Urine calibration standards (N = 10) included CHMC at the following concentrations: 0.49, 0.98, 1.95, 3.91, 7.81, 15.63, 31.26, 62.52, 125.04, and 250.07 ng/mL. Aqueous calibration standards to be used for to method validation tests were prepared by adding 20 µL of each spiking calibration standard into 980 µL of 10 mmol sodium acetate in deionized water:acetonitrile (65:35 v/v) solution. The stock solution and calibration standards were stored at −20 °C until further use.

### Enzymatic hydrolysis of urine samples

Urine standards or samples (1 mL) were added to 15 mL polypropylene centrifuge tubes and then hydrolyzed by adding 400 µL of IMCSzyme® rapid hydrolysis buffer and 200 µL of IMCSzyme® beta-glucuronidase reagent [[2], [24]]. Samples were shaken (35 rpm) for 30 min using an Edvotek® 10 L digital shaking water bath (45 °C) (Edvotek®, Washington, DC). Following glucuronidase hydrolysis, 1.6 mL of 100 mmol ammonium acetate in deionized water (pH 9.7) was added to each sample. The samples were vortexed and centrifuged at 2000 RCF for 10 min in a Cole-Parmer MS3400 centrifuge (Cole-Parmer, Vernon Hills, IL) to precipitate macromolecules.

### Solid-phase extraction

Solid-phase extraction (SPE) was carried out using Strata-X-A 33 µm polymeric strong anion-exchange tubes (200 mg / 3 mL capacity) (Phenomenex, Torrance, CA) housed in a Fluka SPE 16-port vacuum manifold (Honeywell International Inc., Charlotte, NC). Prior to extraction, Strata-X-A tubes were pre-conditioned with 2 mL of methanol and then 2 mL of 100 mmol ammonium acetate in deionized water. The supernatant from centrifugation was transferred from each 15 mL polypropylene centrifuge tube to pre-conditioned Strata-X-A tubes and then washed with 2 mL of 100 mmol ammonium acetate in deionized water. Next, the tubes were washed with 2 mL of methanol and then dried under vacuum for 5 min. The samples were eluted with 3 mL of 5 % formic acid in methanol. The eluants were collected in 15 mL polypropylene centrifuge tubes and then dried under nitrogen gas (10 psi) in a water bath (50 °C) using a TurboVap® LV automated solvent evaporation system (Biotage, Upsala, Sweden). The dried samples were reconstituted in 500 µL of 10 mmol sodium acetate in deionized water:acetonitrile (65:35 v/v) solution, vortexed, and then transferred to 2 mL glass autosampler vials prior to analysis.

### Chromatographic and fluorescence conditions

Fluorescence of CHMC from extracted human urine was measured with an Agilent 6410A fluorescence detector (FLD) coupled to an Agilent 1100 high-performance liquid chromatography (HPLC) system (HPLC-FLD) (Agilent Technologies, Palo Alto, CA). Chromatographic separations were carried out using a Phenomenex Security Guard C18 guard column (4 × 2 mm) coupled to an Agilent Zorbax Eclipse XDB-C8 analytical column (5 µm, 4.6 × 150 mm). Mobile phase A consisted of 10 mmol sodium acetate in deionized water and mobile phase B consisted of acetonitrile. Samples (10 µL) were eluted at 0.8 mL/min through the guard and analytical columns (column oven, 30 °C) with the linear gradient program: 70 % A for 2 min, 70 % A to 10 % A over 8 min, 10 % A for 4 min, 10 % A to 70 % A over 0.5 min, and 70 % A for 2.5 min. Analyte detection was achieved by fluorescence detection (λ_ex_ 355 nm excitation / λ_em_ 460 nm emission) [[2], [26]].

### Calibration curves

Calibration curves for the spiked urine samples and for the aqueous analytical standards were generated from the average response factor of the CHMC fluorescence response areas divided by the respective CHMC concentrations [27,28]. Calibration curve preparation included a control blank with no CHMC standard to check for contamination. The calibration curves met the acceptance criteria if the back-calculated concentrations were within ±15 % of the nominal values of each calibration standard and within ±20 % of the nominal value of the lowest-level calibration standard [23].

### Quality control standards

Urine samples collected from CFTEP technicians were divided into two separate batches and each batch included three levels of quality control (QC) standards (low = 1.95 ng/mL; medium = 31.26 ng/mL; high = 125.04 ng/mL) prepared in duplicate in pooled control urine (N = 6). Additional QC standards were prepared to run concomitantly with method validation tests. The accuracy of the QC standards met the acceptance criteria if the average of the back-calculated concentrations were within ±20 % of the nominal value of the low QC standard and ±15 % of the nominal values of the medium and high QC standards [23]. If the acceptance criteria were not met, the batch was reprocessed.

### Sample stability

To assess stability of CHMC in aqueous solution, two sets of calibration standards were prepared for a short-term stability test at room temperature and a long-term stability test in cold storage. For the short-term stability test at room temperature (23 °C), five replicates of the three levels of QC standards (N = 15) were prepared in 10 mmol sodium acetate in deionized water:acetonitrile (65:35 v/v) solution to be analyzed over a period of 72 h. Each level of standard was analyzed immediately after preparation (denoted as Hour 0), and the remaining standards were kept in the laboratory fume hood. The remaining four replicates of each standard were analyzed at 4, 24, 48, and 72 h.

For the long-term stability test, eight replicates of the three levels of QC standards (N = 24) were prepared in 10 mmol sodium acetate in deionized water:acetonitrile (65:35 v/v) solution to be analyzed over a period of 30 days. Each level of standard was analyzed immediately after preparation (denoted as Day 0), and the remaining standards were stored in a refrigerator (4 °C). Standards were removed from the refrigerator on days 1, 4, 7, 11, 15, 22, and 30, allowed to equilibrate to room temperature for 30 min, and analyzed by HPLC-FLD.

### Detection limits

The method detection limit (MDL) was determined by following the US EPA Definition and Procedure for the Determination of the Method Detection Limit, Revision 2 [29]. The MDL for spiked samples (MDL_s_) was determined by preparing 7 replicates of a low-level calibration standard (0.12 ng/mL) spiked into pooled control urine. The MDL for method blanks (MDL_b_) was determined by preparing 7 replicates of pooled control urine blanks. The replicate spiked samples and method blanks were processed and analyzed following the sample treatment steps outlined previously. The MDL_s_ and MDL_b_ were calculated with the following equation (MDL_x_ = *t*_(n-1)_ × S_x_), where *t*_(n-1)_ is the Student's *t*-value (*t* = 3.143) for degrees of freedom of the replicates (n-1 = 6) and S_x_ is the standard deviation of the calculated concentrations of the spiked samples or the method blanks. The higher value between the MDL_s_ and MDL_b_ was chosen as the MDL [29]. The limit of quantitation (LOQ) was calculated using the equation (LOQ = 10 × S_x_), and the upper limit of quantitation (ULOQ) was considered the highest calibration standard (250.07 ng/mL) [23].

### Accuracy, precision, recovery, and matrix factor

Five replicates of each of the three QC standards were prepared (N = 15) to assess the accuracy, precision, recovery, and matrix factor of the sample treatment method. The standards were spiked into pooled control urine and processed following the sample treatment steps outlined previously. Accuracy of the method met the acceptance criteria if the average of the back-calculated concentrations were within ±20 % of the nominal value of the low QC standard and ±15 % of the nominal values of the medium and high QC standards [23]. Precision of the method met the acceptance criteria if the relative standard deviation (RSD) of the five replicates was within 20 % for the low QC standard and within 15 % for the medium and high QC standards [23].

To assess recovery of the sample treatment method, five replicates of the three QC standards were prepared by spiking 20 µL of the calibration standard into 980 µL of 10 mmol sodium acetate in deionized water:acetonitrile (65:35 v/v) solution. The aqueous QC standards were then analyzed by HPLC-FLD. The recovery of the method was calculated by dividing the average concentration of each level of extracted QC standard by the average concentration of each level of aqueous QC standard [23]. The matrix factor was calculated by dividing the average fluorescence peak response of each level of extracted QC standard by the average fluorescence peak response of each level of aqueous QC standard [23].

## Method validation

**Fig. 2:**
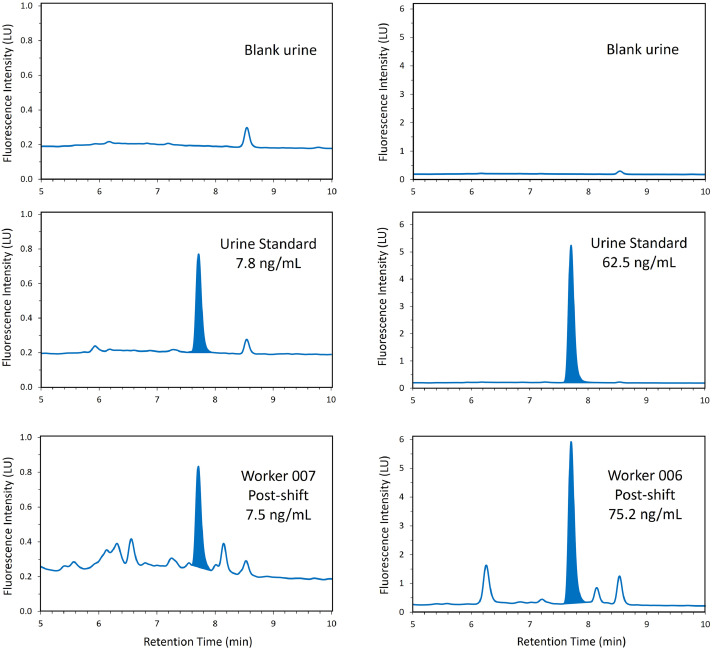
Representative chromatograms of control urine blanks, CHMC standards spiked into control urine prior to sample preparation (7.81 ng/mL on left; 62.52 ng/mL on right), and two urine samples obtained from workers with calculated CHMC concentrations of 7.46 ng/mL (on left) and 75.24 ng/mL (on right).

The HPLC-FLD chromatograms of CHMC fluorescence for a control urine blank, a control urine calibration standard spiked with 7.81 ng/mL CHMC, and a worker post-shift urine sample with a calculated concentration of 7.5 ng/mL CHMC are shown in Fig. 2. The retention time of CHMC with the described HPLC gradient method was approximately 7.7 min. The baseline fluorescence noise at λ_ex_ 355 nm excitation / λ_em_ 460 nm emission was approximately 0.2 luminescence units (LU) in both pooled control urine and in workers’ urine samples for the duration of the LC gradient program.

The calculated average response factor for the calibration curve prepared in pooled control urine was 0.5 with a range of 0.46 – 0.56 and an RSD of 6.9 %. The calibration curve was linear from 0.49 – 250.07 ng/mL (N = 10) with a correlation coefficient of *r* = 0.9988 (CORREL function in Microsoft Excel). The calculated average response factor for the calibration curve prepared in 10 mmol sodium acetate in deionized water:acetonitrile (65:35 v/v) solution was 0.5 with a range of 0.48 – 0.56 (N = 10), an RSD of 6.3 %, and a correlation coefficient of *r* = 0.9997. The calculated MDL_s_ and MDL_b_ were 0.06 ng/mL and 0.03 ng/mL, respectively. Therefore, 0.06 ng/mL was chosen as the MDL. The S_s_ of 0.02 ng/mL for the MDL_s_ replicate spiked samples was used to calculate the LOQ of 0.20 ng/mL. Because the calculated LOQ was lower than the calibration curve range, the lowest level calibration standard was treated as the lower limit of quantitation (LLOQ = 0.49 ng/mL) [23].

**Fig. 3:**
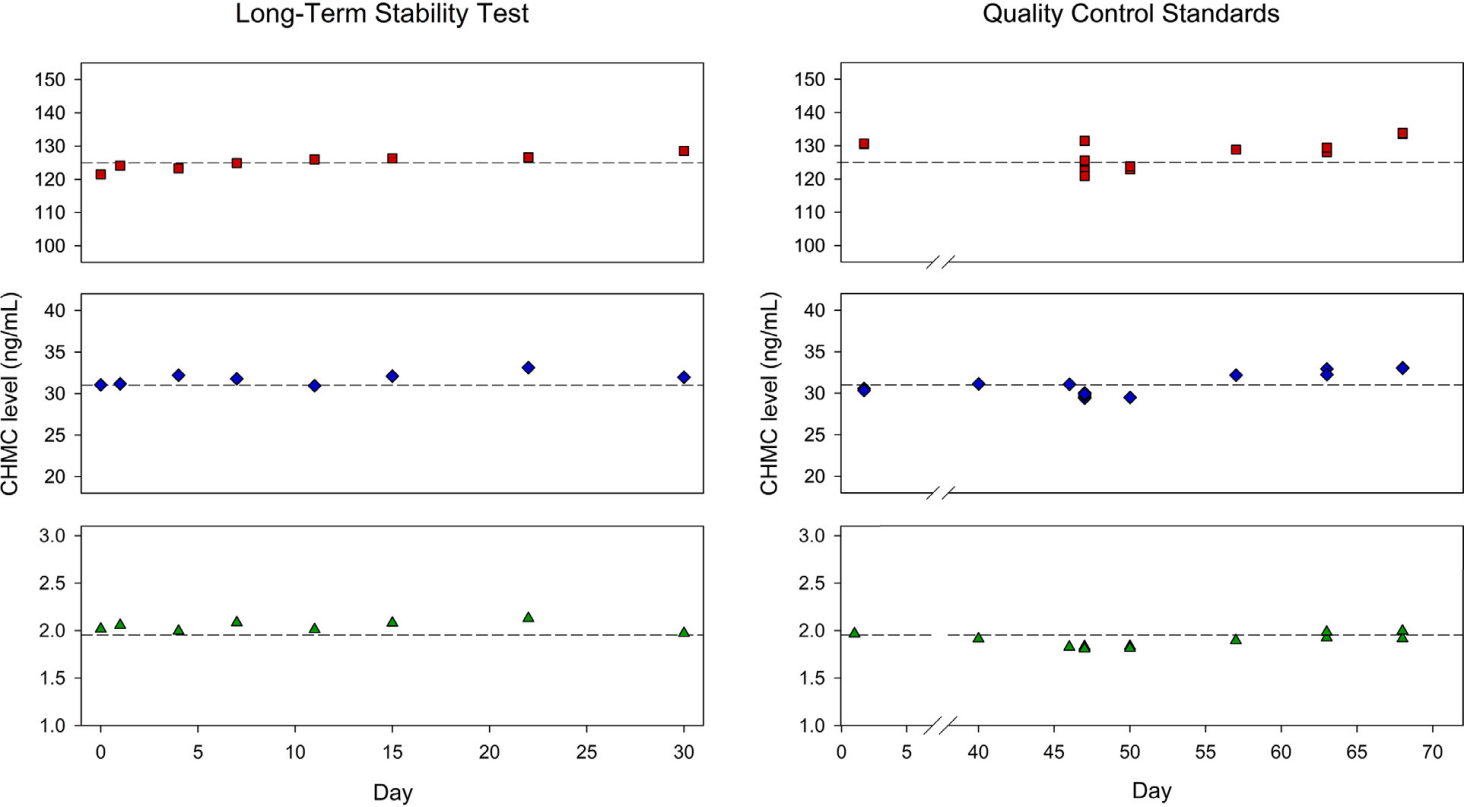
Time-course plots of the three levels of calibration standards (1.95, 31.26, and 125.04 ng/mL) used for long-term storage stability at 4 °C in 10 mmol sodium acetate in deionized water:acetonitrile (65:35 v/v) solution and quality control standards spiked into control urine samples. Urine samples prepared for accuracy, precision, recovery, and matrix factor tests were included on Day 47 of the Quality Control Standards plots.

The results from the method validation tests are summarized in Table 1. The standards prepared in 10 mmol sodium acetate in deionized water:acetonitrile (65:35 v/v) solution for the short-term stability test over a 72-h period at room temperature (see **Supplemental Figure S1**) and for the long-term stability test over a 30-day period at 4 °C were within ±9 % of the expected concentrations (see Fig. 3). The same concentrations chosen for the stability studies were spiked into urine samples and processed following the sample treatment and analytical method for the accuracy, precision, recovery, and matrix factor tests. The accuracy of the CHMC standards spiked into urine was within ±8 % of the expected concentrations with precision of the method ranging from 0.53 – 3.20 %. The average extraction recovery from spiked urine samples for the three concentration levels ranged from 93.3 – 100.1 % with average matrix factors ranging from 0.96 – 1.01. The three levels of CHMC concentrations used for the method validation tests were also prepared separately in control urine as QC samples for two batches of workers’ urine samples and for detection limit tests. The QC standards and workers’ urine were prepared and analyzed concomitantly over the course of 68 days (Fig. 3). The average absolute errors and RSDs of the three levels of QC standards were 97.9 % (3.4 %) for 1.95 ng/mL, 100.5 % (4.3 %) for 31.26 ng/mL, and 103.2 % (2.9 %) for 125.04 ng/mL. All QC standards prepared were within ±8 % of expected concentrations.

**Table 1:** NIOSH method evaluation criterion for three CHMC calibration levels spiked into 10 mmol sodium acetate in deionized water:acetonitrile (65:35 v/v) solution or spiked into control urine samples and processed with the sample treatment and analytical method.

Standard Level		Low	Medium	High
**Concentration**	**N**	**1.95 ng/mL**	**31.26 ng/mL**	**125.04 ng/mL**
Short-Term Stability	5	105.5 %	101.7 %	96.6 %
Long-Term Stability	8	104.5 %	101.7 %	100.1 %
Accuracy	5	93.3 %	95.1 %	100.1 %
Precision	5	0.6 %	0.9 %	3.2 %
Recovery	5	97.3 %	96.5 %	100.8 %
Matrix Factor	5	0.97	0.96	1.01
Quality Control^a^	9–11	92.9 – 102.2 %	94.4 – 105.8 %	98.3 – 107.1 %

N = number of samples prepared per standard level for each method validation parameter. Eleven samples were prepared for QC levels 1.95 and 31.26 ng/mL, and nine urine samples were prepared for QC level 125.04 ng/mL.

a Two additional 1.95 and 31.26 ng/mL QC standards were prepared concurrently with method detection limit standards.

### Urine CHMC levels in CFTEP participants

Urine samples (N = 56) collected from CFTEP participants (*n* = 19) were processed and analyzed following the sample treatment steps described previously. Calculated urine CHMC levels (see **Supplemental Table S1**) were reported to USDA APHIS and FOH. If CHMC was measured below the MDL in a worker's urine sample, the result was reported as a non-detect (ND) [23]. If CHMC was measured between the MDL and the LLOQ, the result was reported numerically in parentheses with one less significant digit [23]. If CHMC was measured above the ULOQ, the urine sample was diluted by a factor of 10 by adding 100 µL urine into 900 µL deionized water and processed and analyzed following the steps described previously. The back-calculated concentration of the diluted urine sample was then multiplied by ten to obtain the original concentration.

**Fig. 4:**
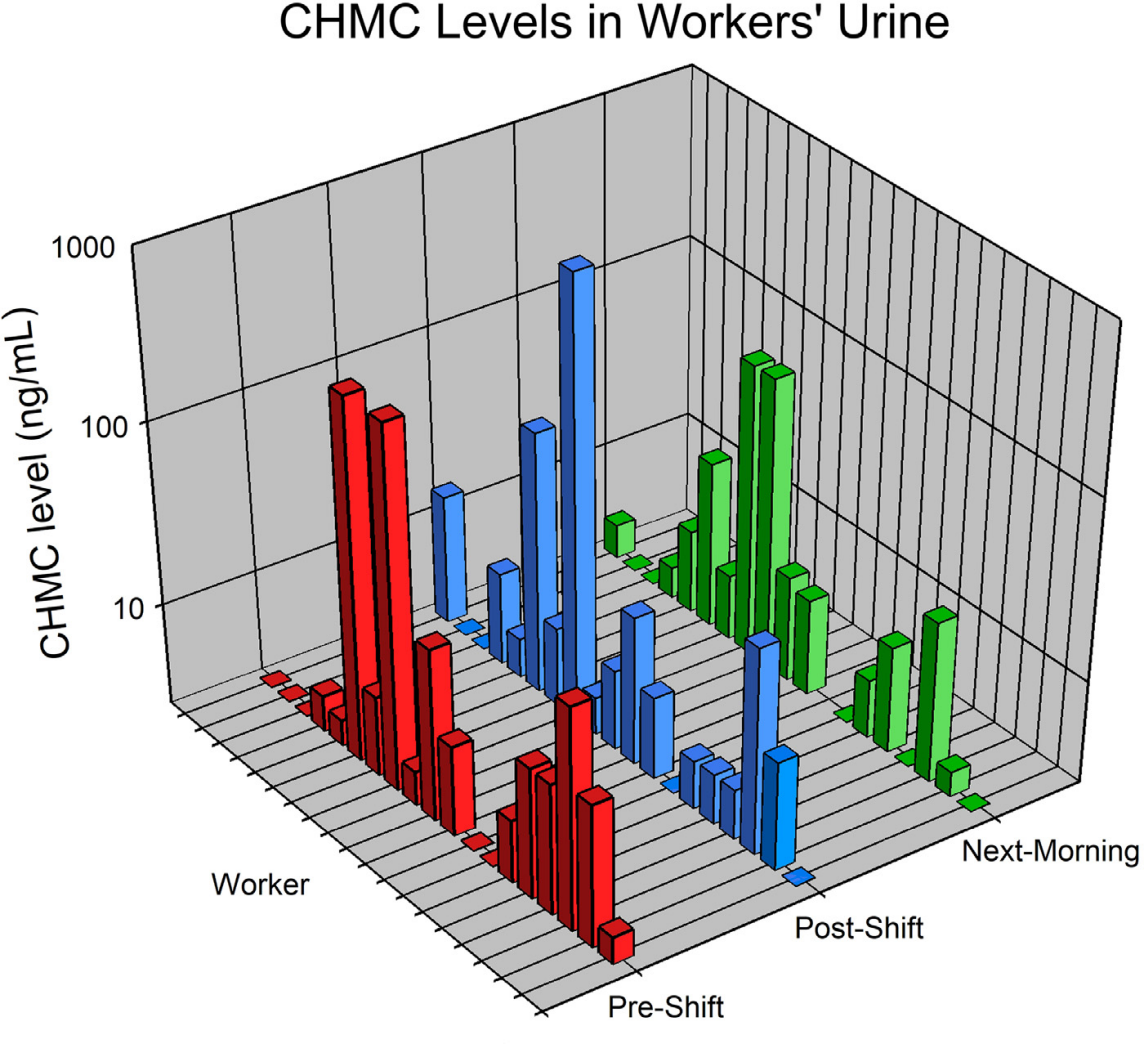
CHMC levels (ng/mL) in urine samples (N = 56) collected pre-shift, post-shift, and next-morning from CFTEP study participants (*n* = 19). The CHMC levels are displayed on a logarithmic scale (log_10_[CHMC]) to account for the wide range of CHMC concentrations measured in the urine samples.

Fig. 4 displays the CHMC levels on a logarithmic scale (log_10_[CHMC]) in pre-shift, post-shift, and next-morning urine samples collected from CFTEP participants. The calculated urine CHMC levels stratified by sample collection shift and the summary statistics can be found in the **Supplemental** (see **Table S1** and **Table S2)**. CHMC was measured above the LLOQ in more than 80 % of all urine samples collected. The highest CHMC level observed was 708.55 ng/mL in a post-shift urine sample, however, the next two highest levels were observed in pre-shift urine samples. Because urine was collected from some workers on Tuesday and Wednesday, they likely were exposed to coumaphos on the previous day.

### Limitations

The purchased pooled control urine had minimal background noise at λ_ex_ 355 nm excitation / λ_em_ 460 nm emission with small peaks at 6.0 min and 8.5 min (see Fig. 2). However, some workers’ urine samples had significant background near the CHMC fluorescence peak and incorporating an experimental or analytical internal standard was not feasible. A stable isotope labelled (SIL) standard of CHMC was not commercially available and was cost-prohibitive to custom synthesize. Furthermore, an SIL standard that co-elutes with the target analyte and has an identical fluorophore will be indistinguishable from the target analyte with fluorescence detection. Identifying a similarly structured organophosphate pesticide metabolite to use as an internal standard proved challenging because of the presence of multiple unknown peaks within 2 min of the CHMC fluorescence peak. We did not test internal standards that may co-elute before 6 min with naturally fluorescent substances in urine [[30], [31], [32]]. Two compounds with intrinsic fluorescence similar to CHMC were tested for use as internal standards. Both 7‑hydroxy-4-methycoumarin (HMC, CAS No 90–33–5) and 6‑chloro-7‑hydroxy-4-methylcoumarin (6CHMC, CAS No 19,492–02–5) were found to co-elute with other substances in the workers’ urine that fluoresce under method conditions. HMC is an environmental breakdown product of coumaphos [33] and was detected in multiple workers’ urine samples without addition of the pure HMC standard. The source of 6HMC in workers’ urine is unknown, but it is likely a product of environmental and metabolic breakdown of coumaphos or HMC. Because we could not find a structurally similar standard that did not coelute with other substances in the workers’ urine, we chose to forego the inclusion of an internal standard.

## Disclaimer

The findings and conclusions in this report are those of the authors and do not necessarily represent the official position of the National Institute for Occupational Safety and Health (NIOSH), Centers for Disease Control and Prevention (CDC) or Federal Occupational Health (FOH) in the Program Support Center (PSC). Mention of any company or product does not constitute endorsement by NIOSH/CDC or FOH/PSC.

## Ethics statements

Human subject institutional review board (IRB) research protocol and urine sample collection was managed by USDA APHIS and FOH researchers. Urine samples were sent to NIOSH blinded with no personal identifiable information included.

## Funding

This work was supported by the 10.13039/100000125National Institute for Occupational Safety and Health, Cincinnati, OH.

## CRediT authorship contribution statement

**Zachary G. Robbins:** Project administration, Methodology, Data curation, Formal analysis, Writing – original draft, Visualization. **Cynthia A. Striley:** Conceptualization, Funding acquisition, Project administration, Investigation, Methodology, Data curation, Validation, Writing – original draft, Visualization. **Lee Wugofski:** Conceptualization, Resources, Writing – review & editing.

## Declaration of competing interest

The authors declare that they have no known competing financial interests or personal relationships that could have appeared to influence the work reported in this paper.

## Data Availability

Data will be made available on request.

## Appendix Supplementary materials

Image, application 1
