# Supplementary material for: Quantification of 3‑chloro-7‑hydroxy-4-methylcoumarin (CHMC) in urine as a biomarker of coumaphos exposure by high-performance liquid chromatography-fluorescence detection (HPLC-FLD)
Source: MethodsX. 2025 Jan 13;14:103171. doi: 10.1016/j.mex.2025.103171 (PMC11791304; doi:10.1016/j.mex.2025.103171)
Supplement: Supplementary file 1 [file mmc1.docx]

**OPTIONAL**

**Supplemental**

*Cholinesterase levels in CFTEP participants*

Red blood cell acetylcholinesterase (RBC AChE) and plasma pseudocholinesterase (PChE) levels were monitored monthly [1] in this worker population by Federal Occupational Health (FOH) in collaboration with the U.S. Department of Agriculture (USDA) Animal and Plant Health Inspection Service (APHIS) Cattle Fever Tick Eradication Program (CFTEP) [2-4]. Cholinesterase inhibition/inactivation is measured as a percentage of the cholinesterase baselines at time of hiring, or by establishing baseline levels after an extended period away from organophosphate pesticide exposures [3]. The RBC AChE and PChE inhibition/inactivation levels before and after the urine collection for 14 of the 19 CFTEP participants were provided for this study. The urine CHMC levels and RBC AChE and PChE levels for each participant are shown in **Table S1**. Cholinesterase levels as a ratio to the baseline cholinesterase levels were provided by FOH for the month of and the month following urine sampling. Cholinesterase levels reported in **Table S1** are averaged because the relative standard deviations of the cholinesterase levels were less than 10 % for all 14 participants. **Table S2** displays summary statistics of urine CHMC levels shown in **Table S1** for all 19 CFTEP participants stratified by pre-shift, post-shift, and next-morning urine sample collection.

**Table S1**. Workplace and biological sample collection for CFTEP study participants (*n* = 19) exposed to coumaphos. The cholinesterase levels are shown as ratios to the baseline cholinesterase levels measured months prior to working with coumaphos.

|  |  |  | **Urine CHMC Level (ng/mL)** | | | **Cholinesterase Level** | |
| --- | --- | --- | --- | --- | --- | --- | --- |
| Location | Worker | Weekday | Pre-Shift | Post-Shift | Next-Morning | RBC AChE | PChE |
| Site 1 | 001 | Monday | 1.22 | 14.10 | 4.19 | 0.989 | 0.867 |
|  | 002 | Monday | (0.2) | (0.1) | (0.4) | 1.017 | 1.165 |
|  | 003 | Monday | (0.3) | (0.3) | 1.81 | - | - |
|  | 016 | Monday | <MDL | <MDL | (0.1) | - | - |
| Site 2 | 004 | Monday | 4.30 | 8.78 | 4.02 | 0.848 | 1.024 |
|  | 005 | Tuesday | 3.79 | 4.49 | 7.77 | 0.993 | 0.828 |
|  | 006 | Tuesday | 263.84 | 75.24 | 22.47 | 0.977 | 0.610 |
|  | 007 | Tuesday | 7.52 | 7.46 | 6.19 | 1.175 | 0.750 |
|  | 008 | Tuesday | 269.48 | 708.55 | 108.48 | 0.886 | 0.676 |
|  | 009 | Tuesday | 4.21 | 4.37 | ND | 0.948 | 0.756 |
|  | 010 | Tuesday | 24.59 | 7.54 | 10.40 | 0.922 | 0.761 |
|  | 011 | Tuesday | 8.65 | 18.08 | 9.25 | 1.037 | 1.153 |
| Site 3 | 012 | Tuesday | 1.31 | 7.76 | - | 1.027 | 1.145 |
| Site 4 | 013 | Tuesday | (0.2) | 0.54 | 1.19 | - | - |
| Site 5 | 014 | Wednesday | 6.11 | 5.04 | 5.70 | - | - |
|  | 015 | Wednesday | 14.60 | 5.16 | 10.58 | 0.951 | 1.111 |
|  | 017 | Wednesday | 46.44 | 38.09 | 21.21 | 0.937 | 0.823 |
|  | 018 | Wednesday | 16.93 | 10.97 | 3.73 | 0.880 | 0.742 |
|  | 019 | Wednesday | 3.85 | 1.58 | 0.75 | - | - |

CHMC = 3-chloro-7-hydroxy-4-methylcoumarin; RBC AChE = red blood cell acetylcholinesterase; PChE = plasma pseudocholinesterase; ND = non-detect; <MDL = calculated CHMC concentration below the method detection limit; (x) = calculated CHMC concentration between the method detection limit and the lower limit of quantitation.

**Table S2**. Summary of CHMC levels in urine collected from CFTEP study participants (*n* = 19) exposed to coumaphos.

|  | **Samples** | | |  | | **Urine CHMC levels (ng/mL)** | | | |
| --- | --- | --- | --- | --- | --- | --- | --- | --- | --- |
|  | N | N > LLOQ (%) |  | | Range | | AM (SD) | GM (GSD) |  |
| **Urine Collection** |  |  |  | |  | |  |  |  |
| Pre-shift | 19 | 15 (79 %) |  | | <LLOQ – 269.48 | | 35.62 (82.20) | 3.14 (17.39) |  |
| Post-shift | 19 | 16 (84 %) |  | | <LLOQ – 708.55 | | 48.30 (160.87) | 3.99 (13.80) |  |
| Next-morning | 18 | 15 (83 %) |  | | <LLOQ – 108.48 | | 12.10 (24.94) | 2.64 (10.45) |  |
| Total | 56 | 46 (82 %) |  | | <LLOQ – 708.55 | | 32.36 (105.35) | 4.00 (14.72) |  |
|  |  |  |  | |  | |  |  |  |

CHMC = 3-chloro-7-hydroxy-4-methylcoumarin; N = number of samples; LLOQ = lower limit of quantitation; AM = arithmetic mean; SD = arithmetic standard deviation; GM = geometric mean; GSD = geometric standard deviation.

**
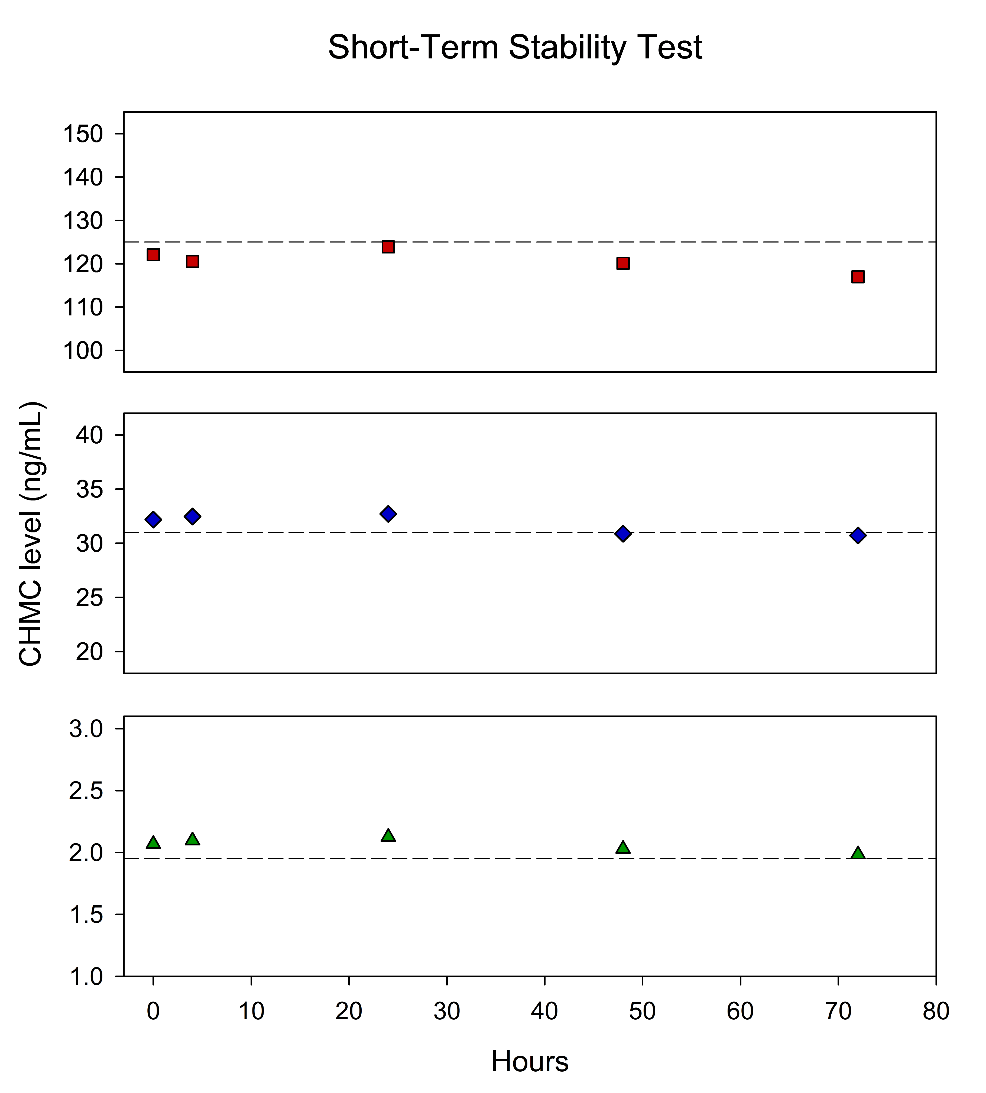
**

**Figure S1**. Time-course plots of the three levels of calibration standards (1.95, 31.26, and 125.04 ng/mL) used for short-term storage stability at room temperature in 10 mmol sodium acetate in deionized water:acetonitrile (65:35 v/v) solution.

**References**

[1] Quest Diagnostics, Cholinesterase, RBC and Plasma, Test Code 338, CPT Code(s) 82480, 82482 (2019) (Accessed on September 19, 2024). <https://testdirectory.questdiagnostics.com/test/test-detail/338/cholinesterase-rbc-and-plasma?cc=MASTER>.

[2] US Environmental Protection Agency (EPA), Reregistration Eligibility Decision (RED) Addendum and FPQA Tolerance Reassessment Progress report: Coumaphos, EPA 738-R-00-010, 2000 September 2000. <https://www3.epa.gov/pesticides/chem_search/reg_actions/reregistration/tred_PC-036501_1-Sep-00.pdf>.

[3] G.A. Thomas, L.J. Delaney, C. Mueller, E. Page, Evaluation of coumaphos exposure among tick eradication workers, J. Occup. Environ. Med. 52 (2) (2010) 131–136, doi:10.1097/JOM.0b013e3181cd7e80.

[4] US EPA, Coumaphos Draft Human Health Risk Assessment For Registration Review, EPA-HQ-OPP-2008-0023-0023, 2016. <https://www.regulations.gov/document/EPA-HQ-OPP-2008-0023-0023>.
